# Dog and Cat Interactions in a Remote Aboriginal Community

**DOI:** 10.3390/ani8050065

**Published:** 2018-04-26

**Authors:** Brooke Kennedy, Wendy Y. Brown, Karl Vernes, Gerhard Körtner, James R. A. Butler

**Affiliations:** 1 Canine and Equine Research Group, University of New England, Armidale, NSW 2351, Australia; wbrown@une.edu.au (W.Y.B.); james.butler@csiro.au (J.R.A.B.); 2 Ecosystem Management, University of New England, Armidale, NSW 2351, Australia; kvernes@une.edu.au; 3 Zoology, University of New England, Armidale, NSW 2351, Australia; gkoertne@une.edu.au; 4 CSIRO Land and Water, GPO Box 2583, Brisbane, QLD 4001, Australia

**Keywords:** dog, cat, interactions, remote Aboriginal community

## Abstract

**Simple Summary:**

Australian remote Aboriginal communities commonly have large, free-roaming dog populations and relatively small cat populations. However, cats are becoming increasingly popular pets in these communities and it is essential to determine their potential impacts on wildlife to inform animal management practices. In a small island community, this study provided baseline population demographics and investigated dog and cat roaming behaviours and interspecific interactions. The dog population, estimated at 343 dogs, showed active periods at dawn and dusk. The cat population had doubled in 6 months to approximately 83 cats. Cats were observed roaming mainly from dawn until dusk, possibly avoiding periods when dogs were most active. The majority of cats were observed nonroaming. Three cats, however, were captured roaming during the night when all flightless wildlife were observed on our camera traps, suggesting potential impact on local wildlife through their hunting activities. These baseline data provide evidence on which to base management programs that include cats and their impacts on native wildlife. Further research using the multiple methods piloted in this study are warranted to monitor dog and cat populations and their interactions in this island community.

**Abstract:**

This study examined dog and cat demographics, roaming behaviours, and interspecific interactions in a remote Aboriginal island community using multiple methods. Our results revealed temporal differences between the roaming behaviours of dogs, cats, and wildlife. Dogs showed crepuscular behaviour, being active around dawn (5:30 a.m. to 9:30 a.m.) and dusk (6:00 p.m. and 11:35 p.m.). The majority of cats were active between dawn (6:30 a.m.) and dusk (7:30 p.m.) and travelled shorter distances than dogs. However, some cats were also observed roaming between dusk and dawn, and were likely to be hunting since flightless wildlife were also recorded on our remote-sensing cameras during this time. These baseline data provide evidence to suggest that new management programs are needed to reduce the number of roaming cats and therefore their potential impacts on native wildlife. Collaborations between Aboriginal owners and other stakeholders is necessary to design innovative and effective animal management and policy on the island.

## 1. Introduction

Owned dog (*Canis familiaris*) populations in remote Aboriginal communities (RACs) in Australia have been estimated at 50–62 dogs per 100 people, much greater than the overall Australian average of 16 dogs per 100 people [1]. Furthermore, dogs in RACs are generally free-roaming (FRD). Consequently, dogs in RACs have received increasing attention in the scientific literature. By comparison, the estimated population of domestic cats (*Felis catus*) in RACs is 7–9 cats per 100 people, which is lower than the overall Australian average of 11 cats per 100 people, and there has been little research published on this issue. However, as cats are becoming more popular pets in RACs, research is needed to determine their potential environmental impacts and inform animal management practices.

The risk of zoonotic transmission increases when dogs and cats are kept at high population densities in association with humans [2], such as in RACs. Sarcoptic mange (*Sarcoptes scabiei*), hookworm (*Ancylostoma caninum*), fleas, ticks (*Rhipicephalus sanguineus*), and giardia (*Giardia duodenalis*) have all been documented in dogs in RACs [3,4,5,6]. Limited research conducted on cats in Aboriginal communities has identified tapeworm (*Spirometra erinacei*), *Oncicola* spp., hookworm (*Ancylostoma caninum*), and *Toxoplasma gondii* in cats in Northern Australia [4]. In communities with a high prevalence of giardia in dogs, and hookworm and toxoplasma in cats, these parasites were also found in the children and adults living there. This is of concern for public health, particularly for children under 5 years of age [3]. Domestic animals can also impact native wildlife. Globally, FRDs threaten wildlife conservation through predation [7,8,9], disturbance [10,11,12], competition [13,14], and disease transmission [3,4,6,15,16]. Cats primarily impact wildlife through direct predation, but also competition and disease transmission [17]. The mainland small and medium mammal decline in Australia is suggested to be largely due to environmental change associated with the loss of Aboriginal fire management practices, or an interaction between that and exotic predators (i.e., red fox, (*Vulpes vulpes*) and cats) [18]. Many native island species have reduced defences against mammalian predators; feral cats on islands alone are responsible for at least 14% of global animal extinctions and the main threat to almost 8% of critically endangered birds, mammals, and reptiles [19]. Recent studies on the Tiwi Islands have shown a decline in the populations of the threatened brush-tailed rabbit rat (*Conilurus penicillatus*), and revealed that they are restricted to areas where feral cat detection is low and shrub density is high [20].

Dogs are also known to have made contributions, whether known or potential, to the extinctions of 11 vertebrate species and the threatened status of 188 others: 96 mammals, 78 birds, 22 reptiles, and 3 amphibians [21]. Dogs can also severely impact small populations, for example, they are known to have killed 500 out of 900 North Island Brown Kiwis (*Apteryx mantelli*) from their biggest population [8], have been the main agent of local extinction of the Conga huita (*Capromys pilorides*) in Cuba [9], and caused a decrease in the population of Marine Iguanas (*Amblyrhynchus cristatus*) on several Galapagos Islands [7]. Studies focusing on Australia’s decline in mammal fauna tend not to include dogs (domestic dogs owned or wild (*Canis familiaris*), dingoes (*C. dingo*), and their hybrids), as the studies rely on pre-European data for comparisons, however, dingoes are not post-European introductions. Therefore, comparisons must be made post-intervention. The Tiwi Islands are one of only two of the 85 Australian Bioregions with all original mammal species judged to persist in >50% of its former range within the bioregion [17], and with the known and potential impacts on wildlife by cats and dogs, an intervention is crucial.

Dog health programs that include sterilization and parasite control have been introduced to many RACs in an attempt to mitigate poor dog health and overpopulation [5,22], and are also likely to reduce the risks associated with disease spread and impacts on wildlife. With cats becoming popular pets, cats should be included in future animal health programs. It is anticipated that a reduction in the dog and cat populations will limit the impacts they have on native wildlife. This study focused on an island RAC where FRDs are well established and where a dog health program providing sterilization and parasite control has been in place for several years [22]. The recent emergence of pet cats in the community raised concerns about their potential impact on the local wildlife and current animal management practices. In response to these concerns, the present study was undertaken to provide baseline population demographics on community-owned dogs and cats and to investigate their respective roaming behaviours. Multiple methods were tested to investigate dog and cat interactions in RACs and their potential relationships with wildlife.

## 2. Materials and Methods

### 2.1. Study Site

The Tiwi Islands are approximately 60 km north of Darwin and are comprised of 11 islands. The larger two islands, Melville and Bathurst, are the only inhabited islands of the 11 and are 5788 km^2^ and 1693 km^2^ in size, respectively (any further mention of the Tiwi Islands in this paper is in reference to only these two main islands). Open eucalyptus forest (mainly *Eucalyptus miniata*, *E. tetrodonta*, and *Corymbia nesophilia*) covers 76% of the Tiwi Islands. The remaining is made up of mangroves, swamps, open shrub lands, monsoon rainforest, and cleared land where the settlements are located [23]. Wurrumiyanga, the capital and largest community on the Tiwi Islands, is located in the southeast corner of Bathurst Island (11°45′42.99″ S, 130°37′54.48″ E) and is where this study took place. There are two main seasons: the dry season from May to October and the wet season from November to April [23]. Mean minimum and maximum temperatures range from 18.3 to 30.1 °C in July and from 25.0 to 33.2 °C in November [24]. According to the 2016 census [25], the Wurrumiyanga community is home to 1563 people, with 1411 (90.2%) being Aboriginal [25].

### 2.2. Dog and Cat Census

In February 2017, at least one member of the research team and one Tiwi Land Council (TLC) Ranger undertook a door-to-door census. Each household, where someone was home, was asked if they had any pets; if yes, quantity, species, gender, and reproductive status for each pet was recorded. Local knowledge and observations were used to target households that owned cats and data was collected from all these houses. The cat population data were updated at three subsequent time points (April, June, and August 2017). This was completed by the same teams and data were collected from all known cat-owning households.

### 2.3. Roaming Behaviours

#### 2.3.1. Direct Observation—Transect Drives

**Figure 1:**
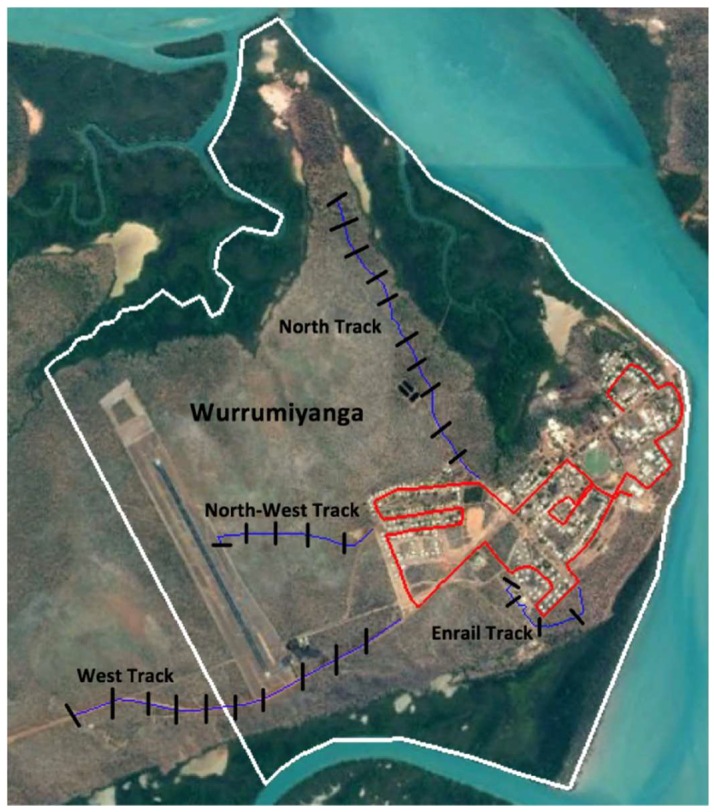
Wurrumiyanga, NT, Australia showing the predetermined transect (red) for direct observations of cats and camera transects (blue) where cameras were placed (black) every 200 m along roads/tracks.

Cat roaming behaviour was monitored directly by driving along a predetermined transect (Figure 1) through the community every 4 h (6 p.m., 10 p.m., 2 a.m., 6 a.m., 10 a.m., 2 p.m.) over 3 days (18–21 April 2017). The transect was 8.24 km in length and covered 75.5% of the community’s roads. Hand-held LED torches were used during the 10 p.m. and 2 a.m. drives in addition to the vehicle’s headlights to aid visibility. Start and finish times were recorded for each drive and a tally sheet was used to record the number of cats observed roaming and nonroaming. A cat was identified as ‘roaming’ if it was observed outside the perimeter of the yard surrounding a house, and ‘nonroaming’ if it was inside a house yard. A second study was conducted over 3 days in June (5–8 June) along the same transect and at the same time points.

#### 2.3.2. Indirect Observation—Remote-Sensing Cameras

In conjunction with the second observation period in June, 29 heat-in-motion camera traps (Scoutguard SG550, Boly Media Communications, China) were installed along four tracks (Figure 1) running in different directions from the community. Single cameras were systematically placed on the right side of the track, when heading out of town, every 200 m starting from the 200 m point (Figure 1). Cameras were placed ~1 m from the road’s edge, on trees, if possible; if no suitable trees were available, star-pickets were placed in the ground. Cameras were vertically positioned 90 cm above track height, with an angle of incidence of 22° to the track and ~5–6 m from the centre of the road. The cameras were set as follows: Passive Infrared (PIR) sensitivity: high, photo captures per trigger: 3, interval between triggers: 0 s and image size: 5 megapixels. Cameras were deployed for seven consecutive nights from 5 June 2017. The dirt tracks were all accessible by foot and vehicle and surrounded by open eucalypt forest. Minimum temperatures ranged from 16.7–21.7 °C and maximum temperatures ranged from 28.3–32.0 °C with no rainfall over the study period [26]. All dogs and cats were able to be individually identified and all events were used. If wildlife were able to be individually identified, all events were also used. However, for wildlife that could not be easily identified (smaller mammals and birds, particularly those not close to the camera), the presence of animals at a camera trap within 30 min of one another were conservatively considered to be nonindependent and part of the same event. The number of events were then standardized across cameras as number of events per night.

#### 2.3.3. Tracking—GPS Loggers

**Figure 2:**
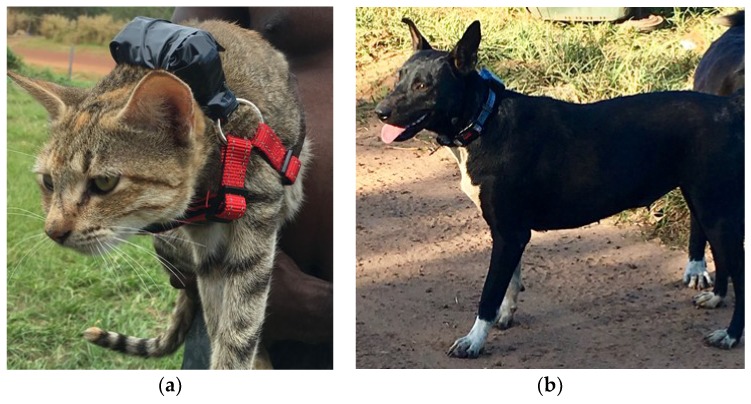
To monitor roaming behaviour and interspecific interaction, igot-U GPS loggers were attached to (**a**) harnesses on cats and (**b**) collars on dogs.

The movements of 15 cats and six dogs were tracked with a Mobile Action (New Taipei City, Taiwan) i-gotU low-cost GPS logger device [27,28] during the second study in June (5–11). Devices were preset to a tracking interval of 15 min (900 s waypoint logging interval and 120 h best battery power). These low-cost GPS loggers do not provide information on the number of satellites or dilution of position (DOP). We therefore filtered the downloaded location records for unrealistic large distances in conjunction with turning angles close to 180° as well as implausible locations (i.e., ocean records). Morris and Conner [27] report that for stationary loggers situated outside with different levels of cover, these loggers are sufficient for studies requiring accuracies of approximately 10 m. Cats and dogs were recruited opportunistically by asking community members during the house-to-house census if they would be willing to participate in the study. Animals were selected that were owned and unrestrained (i.e., free to roam) and participated with the permission of their owners. Each of the cats were fitted with an off-the-shelf small dog harness (Figure 2a) and the dogs with an off-the-shelf dog collar with reflective strip (Figure 2b). Once the harness/collar was fitted, the device was turned on and attached to the collar/harness with electrical tape. A cover (lid of a Hammond Manufacturing_®_ Handheld Enclosure, made of GP ABS material and 50 × 35 × 2 mm in size) was used on top of the device to prevent it from being switched off or damaged. The devices were retrieved at the end of the observation period and data were downloaded.

### 2.4. Data and Statistical Analyses

A one-way ANOVA was used to analyse the effect of roaming during the direct observations (transect drives). Both studies were pooled and a Rayleigh test was used to determine whether the time of the day influenced the roaming behaviour of cats and dogs. A Rayleigh test is a test for randomness in circular data such as time of day [29]. If data distribution is deemed nonrandom, a mean vector is calculated. The direction of the vector indicates the mean time of activity (roaming or nonroaming), while the length of this vector “r” is a measure of dispersion ranging from random (0) to highly clustered (1), as used by Körtner et al. [30].

During the indirect observations (remote sensing cameras), one camera (#38, North track, 200 m) was destroyed by a vehicle and another (#9, Enrail track, 800 m) was moved off the track resulting in lost data at these locations. Camera distances were therefore recorded as distances from the nearest household rather than distance from town along the track. Observation counts verses distance to the nearest house were analysed using a general linear model with a quasi-Poisson error distribution to account for the overdispersion in our dataset [31]. Again, Rayleigh tests were employed to determine the influence of time of day on camera captures.

GPS data were analysed using the methods of Sparkes et al. [28]. After removing unreliable data points (see above), activity ranges (AR) were calculated for each animal as a Minimum Convex Polygon (MCP) and potential differences between cats and dogs were assessed using a one-way ANOVA. Dog 3 made two trips in a vehicle with its owners to another settlement on the island, but although these waypoints were “correct data”, they were deleted from the data set as the dog didn’t have a choice of free movement as he was restrained by the vehicle. In addition to AR, we calculated the distance moved per 24 h (sum of all distances between consecutive waypoints from midnight to midnight), per night (sunset to sunrise), and per day (sunrise to sunset) provided more than 5 data points were available for a particular period. In this case, species differences were assessed using linear mixed-effect models (package nlme for R) with the individual as a random factor to account for the multiple measures for individuals. Furthermore, we separated activity and rest based on the speed the animal moved between subsequent location records. By applying a speed threshold of 2 m/min (dogs) and 1 m/min (cats), we removed more than 50% of the data and the vector direction calculated from the Rayleigh tests had stabilized (i.e., removing more data did not change the direction of the vector nor did it improve vector length). In this context it has to be noted that the calculated speed is not the actual speed of an animal moving, but an integration of all turning angles and speeds over 15 min. Vector averaging was performed according to Zar [29]. We further analysed the proximity of the collared individuals to each other and, taking the accuracy of the GPS loggers into account, used a 20 m threshold as a measure of a potential contact between them [28].

## 3. Results

### 3.1. Dog and Cat Census

All houses in the community were visited at least once, and return visits were made to houses where no occupants were home at the initial visit. Due to time constraints and occupants being away from their homes, a total of 225 houses (out of 344) were surveyed representing 65.4% of the households in Wurrumiyanga. All known households with pet cats were recorded (17/344 in February and 27/344 in August).

The February 2017 census recorded 198 dogs from 105 households (46.7%) and 120 households (53.3%) with no dogs. The male:female ratio was 0.79:1. Just over half the dog population was desexed (55.6%), with 31.8% being entire and 12.6% unknown (owners couldn’t remember, and the dog was free-roaming so we could not confirm). According to the ABS 2016 Census, there is an average of 4 people per household in Wurrumiyanga. This equates to approximately 22 dogs per 100 people. The 2016 census also reported the total population of Wurrumiyanga to be 1563 people which leads to an estimated population of 343 dogs (by extrapolation).

The February 2017 census recorded 41 cats from 17 households (4.9%) and 327 households (95.1%) with no cats. The male:female ratio of cats was 0.66:1. Only 17.1% of the cat population was desexed, 7.3% had an unknown reproductive status leaving 75.6% entire. By the August 2017 census, the cat population had doubled to 83 cats (Table 1). However, as a result of a targeted education and free cat desexing program (8–9 June), 68.7% (n = 57) of the cats were desexed (Table 1).

### 3.2. Roaming Behaviours

#### 3.2.1. Direct Observation—Transect Drives

Study 1 (April 2017) yielded a total of 92 cats recorded during 12.15 h of observation over 3 days (mean time per transect = 40.5 ± 9.4 min). Per transect there were significantly fewer (*p* < 0.001, Table 2) cats (9) roaming than cats (84) nonroaming. Study 2 (June 2017) recorded a total of 55 cats during 12.75 h of observation (mean time per transect = 42.5 ± 7.4 min). Again, there was a significant difference (*p* < 0.001, Table 2) for behaviour type, with fewer cats (9) roaming than cats (46) not roaming. A *t* test revealed that the cats seen per day did not statistically differ between the two studies, although it came close to significance (*t*_3.3_ = 2.89, *p* = 0.056). Perhaps because of the small number of cats observed roaming (n = 18 for the pooled studies), a Rayleigh test could not determine a time preference for this behaviour (*p* = 0.32, Table 3). In contrast, time of day had a significant effect on the number of cats seen not roaming with a mean vector at 12:31 a.m. (*p* < 0.001, Table 3) with most cats having been observed during the 10 p.m. (n = 28) and 2 a.m. (n = 36) surveys.

#### 3.2.2. Indirect Observation—Remote-Sensing Cameras

**Figure 3:**
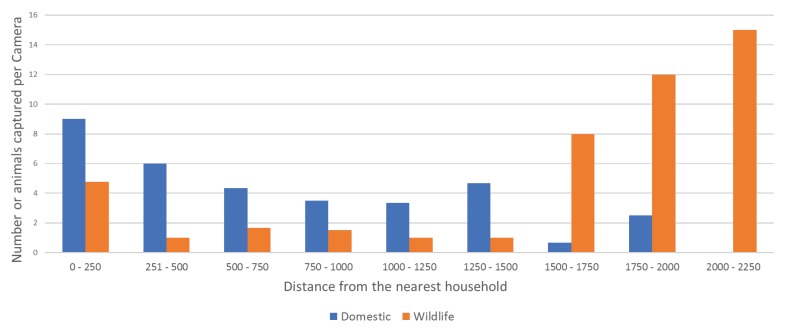
Number of domestic animals (dogs, dogs with owners, and cats) (blue) and other mammals and birds (orange) observed indirectly on remote-sensing cameras against the distance of the camera from the nearest house.

There were 203 independent captures of animals on camera traps across 110 camera days. A total of 242 animals were captured from 11 categories: dog, dog with owner, cat, agile wallaby (*Macropus agilis*), northern brown bandicoot (*Isoodon macrourus*), brushtail possum (*Trichosurus vulpecula*), bush stone-curlew (*Burhinus grallarius*), red-tailed black cockatoo (*Calyptorhynchus banksii*), Australian magpie (*Gymnorhina tibicen*), crested pigeon (*Ocyphaps lophotes*), magpie-lark (*Grallina cyanoleuca*), and unknown (Table A1 in Appendix A). Dogs with owners were defined as captures that contained both dogs and people whether the dogs were leashed or not. Dogs were captured on 58.6% (n = 17) of the cameras across all four tracks and dogs with owners on 17.2% (n = 5) on all but the West track. Cats were captured on 10.3% (n = 3) of the cameras only on the Enrail track and were observed ≤150 m from the nearest house (Table A1). Dogs were captured at ≤1358 m, ≤ 1800 m, ≤792 m, and ≤149 m distance from the nearest house along the north, west, northwest and Enrail tracks, respectively (Table A1). We could not establish a significant relationship between the number of observations and distance to the nearest house (domestic animals; *t* = 1.65, *p* = 0.11; wildlife: *t* = 1.18, *p* = 0.25) (Figure 3).

The timing of camera captures for dogs appeared to show a diametrically bimodal distribution rendering the standard Rayleigh test ineffective. Therefore, an angle doubling procedure was evoked [29]. The Rayleigh test on the transformed data revealed that the dogs roamed along an 8:06 a.m. and 8:06 p.m. axis (Table 2). All cats were captured at night, however, statistical analysis was impossible with only three events. Wildlife tended to be captured at 11:50 p.m. and this changed only slightly to 12:06 a.m. when flying birds, the only daytime wildlife records, were excluded (Table 2).

#### 3.2.3. Tracking—GPS Loggers

Due to faulty devices (n = 5) and five cats removing their harnesses (harnesses were initially too loose), data were collected from four dogs and six cats (Table A2). Dog ARs ranged from 1.23 to 45.72 ha and cat ARs ranged from 0.1 to 2.47 ha with a one-way ANOVA of species effect nearing significance (*p* = 0.057, Table 2). Similarly, over 24 h dogs appeared to travel further than cats, but again the test failed to reach significance (*p* = 0.054, Table 2). However, when analysed separately for day- and nighttime, dogs travelled significantly further than cats (*p* < 0.05, Table 2). Individual data are presented in Table A2.

**Figure 4:**
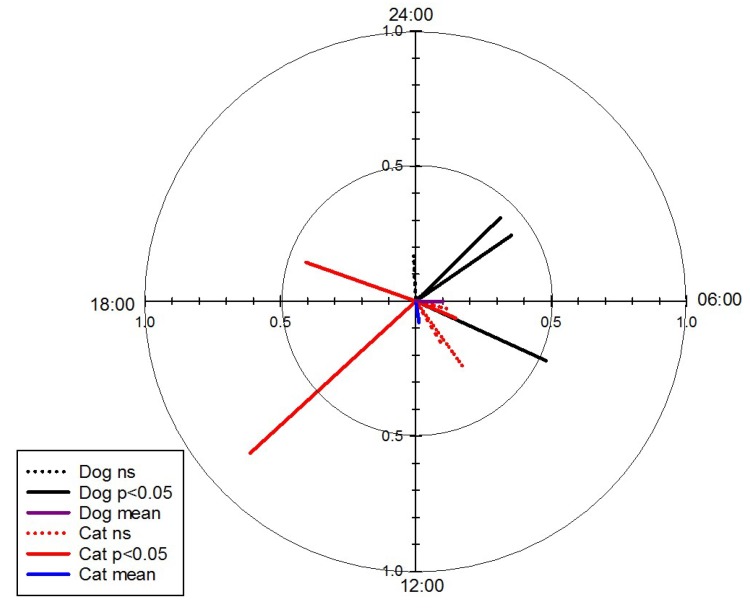
Circular plot of the activity vectors of: all collared cats (red) and the mean cat vector (blue), all collared dogs (black) and the mean dog vector (purple) and the dogs captured on camera traps (green). Solid lines indicate a significant value (*p* < 0.05) whilst dotted lines indicant a nonsignificant value.

Activity records for dogs clustered around 6:02 a.m., whereas cat activity was most prominent at 11:24 a.m. (Table 3). Individual activity vectors are presented in Figure 4 and Table A2. From a total of 1410 GPS fixes (dogs, n = 647 and cats, n = 763), only one contact was recorded between Dog 3 and Cat 4 having been 8.3 m apart from each other within 1.1 min. However, this was despite living directly across the road from each other (15 m between fences, 45 m between front doors).

## 4. Discussion

This is the first study to simultaneously examine the roaming behaviors, interspecific interactions, and demographics of both dogs and cats in a remote Aboriginal community using a variety of methods. There are pros and cons to all the methods used in this study. The direct observations (transect drives) in this study were a quick and low-cost but labour-expensive way to count the number of cats roaming and nonroaming in a population at any one time. However, the disproportionally small number of cats seen roaming could imply that these cats, like their feral cousins, are secretive and difficult to observe when they prowl away from their home yard. Low visibility and the time constraints of the survey design might compromise the effectiveness of the direct observation approach for cats. In comparison, camera transects were more costly even though only 29 cameras were deployed. Nevertheless, they were less labour-intensive, and had the advantage of continuous observations. However, there are many factors that affect capture rates, such as the positioning of the cameras, which can be manipulated to maximize the potential to capture a species of interest [32,33]. In this study, cameras were placed along tracks and positioned to increase the chances of capturing dogs, therefore probably limiting the ability to capture cats and wildlife as indicated by the only three cat records. Future studies may need to consider different camera placements, but it might be near impossible to optimize capture rate for all species of interest at the same time. Nevertheless, for investigating wildlife–cat interaction, this method appears most promising. GPS loggers made for sports activity or pets are a low-cost alternative to GPS collars with or without radio-telemetry. They record continuously all outside locations rather than just where a camera trap is placed. Therefore, habitat use is arguably best studied using this method. However, to extrapolate results to a population level and to study interactions between individuals, a substantially larger sample size is needed. Furthermore, longer-term studies require improvements made to the logger design (e.g., battery capacity), further adding to the complexity and cost for the method. In this study, it was advantageous to use multiple methods, enabling data to be corroborated and the ability to answer different research questions.

Our results revealed temporal differences between the activity patterns of dogs, cats, and wildlife. Based on trail cameras and individual GPS loggers, dogs in this study showed crepuscular or early morning activity, which is consistent with research findings for other FRDs [13] and wild dingoes [34]. As far as cats are concerned, more cats were seen at night, but then mostly in the yards of houses. Trail cameras were less effective, but these suggested, if anything, nighttime roaming behavior, but sample size was too small for statistical analysis. While nighttime records are consistent with the usual hunting behavior of cats, individual GPS records actually showed more daytime activity. However, the locomotion recorded by the GPS loggers is not equivalent to the roaming behavior assessed by the other two methods. Unless hunting away from houses is a very regular occurrence and both cameras as well as direct observations suggest otherwise, other types of locomotion perhaps instigated by the cats’ owners and or community dogs could easily shift the time vector for activity towards the day. The small activity ranges seen and short distances moved confirm that at least the collared cats were basically domestic pets and not feral animals.

The majority of cats roamed between dawn (6:30 a.m.) and dusk (7:30 p.m.) and travelled shorter distances with a trend towards smaller ARs than dogs. However, some cats also roamed between dusk and dawn, and were likely to be hunting since all records of flightless wildlife were captured during this time (6:21 p.m. to 7:07 a.m.), consistent with general knowledge that cats are nocturnal hunters. It is interesting that the majority of the cats in this study were not observed roaming and were not active at night. The small number of interspecific contacts recorded also suggests that cats may have been avoiding dogs, and were therefore most active during the day or night. FRDs are known to prey upon cats [16] (Butler et al. this issue), and therefore it is not surprising that cats avoid dogs, at least temporally. However, the short length of GPS collar attachments on cats (mean 4.5 days) and the few individuals collared limits our confidence in this conclusion. Further longer-term research on a larger number of animals is clearly needed [28].

The total number of cats observed in Study 1 (n = 92) was somewhat higher than that in Study 2 (n = 55), but on a daily basis the two studies did in fact not differ. However, it is obvious that the increase in cat numbers in the community did not result in more cat observations. Seasonal differences in vegetation structure, sightability, and cat behavior between wet and dry season could easily account for the inconsistency between cat numbers and observation, while other explanations such as rate of desexing might also have contributed [20,35,36,37]. The extrapolated dog population of Wurrumiyanga estimate of 22 dogs per 100 people (343 dogs) is almost identical to the 21 dogs per 100 people (326 ± 52 dogs) estimated by Sparkes et al. [28] in 2014. This suggests that the dog population has remained stable over the last three years. Although the population is stable, a new management plan to decrease the overall population may decrease the potential impact on native wildlife. During the dog census undertaken by Brown et al. [22] in 2014, the cat population of Wurrumiyanga was six desexed cats that were house-confined (unpublished data). The population is now 13.5 times higher, and had increased by 2-fold in 6 months. Cats roaming during the wildlife activity period are likely to have detrimental impacts on the local wildlife populations. Direct predation is the major impact feral cats have on Australia’s native fauna [17]. With an estimated 6.3 million cats covering >99.8% of Australia’s land area [38], it is particularly important that cat-free Australian islands are maintained as such. With the Tiwi Islands being one of Australia’s only bioregions to have retained all of its historical mammal species [39], it is critical that they are established as a conservation refuge [40]. To achieve this, management of cats on Northern Australian islands must be developed collaboratively by the Aboriginal owners and other stakeholders [40]. The methods trialed by this study will assist in supporting and monitoring these efforts.

## 5. Conclusions

Our results revealed temporal differences between the roaming behaviors of dogs and cats. Dogs showed crepuscular behavior whilst most cats roamed between dawn and dusk and travelled shorter distances than dogs. This behavior may be to avoid dogs, at least temporally. However, some cats roamed between dusk and dawn as did all flightless wildlife, indicating that the cats may have been hunting. The stable dog population and growing cat population in this remote Aboriginal island community have the potential to impact native wildlife. Our study provides baseline population demographics on the community-owned dogs and cats and their roaming behaviours upon which to base future management plans in consultation with the Traditional Owners and stakeholders. 

## Figures and Tables

**Table 1:** This table shows the population demographics of owned domestic cats and dogs in Wurrumiyanga, Tiwi Islands, NT, Australia collected during a door-to-door census conducted at different time points in 2017.

Sex	Desexed	Entire	Unknown	Total
Dog Census				
February				
Female	74 (71.2%)	23 (22.1%)	7 (6.7%)	104
Male	36 (47.3%)	29 (38.2%)	11 (14.5%)	76
Unknown	0	4 (36.4%)	7 (63.6%)	11
Total	110 (57.6%)	56 (29.3%)	25 (13.1%)	191
Cat Census				
February				
Female	4 (22.2%)	12 (66.7%)	2 (11.1%)	18
Male	3 (25%)	8 (66.7%)	1 (8.3%)	12
Unknown	0	11 (100%)	0	11
Total	7 (17.1%)	31 (75.6%)	3 (7.3%)	41
April				
Female	14 (36.8%)	21 (55.3%)	3 (7.9%)	38
Male	6 (28.6%)	14 (66.7%)	1 (4.8%)	21
Unknown	0	12 (92.3%)	1(7.7%)	13
Total	20 (27.8%)	47 (65.3%)	5 (6.9%)	72
June				
Female	29 (60.4%)	19 (39.6%)	0	48
Male	13 (43.3%)	17 (56.7%)	0	30
Unknown	0	2 (100%)	0	2
Total	42 (52.5%)	38 (47.5%)	0	80
August				
Female	37 (72.5%)	14 (27.5%)	0	51
Male	19 (61.3%)	12 (38.7%)	0	31
Unknown	1 (100%)	0	0	1
Total	57 (68.7%)	26 (31.3%)	0	83

**Table 2:** Summary statistics for transect observations and GPS tracking. Transect observations are presented as the mean number of cats observed where n = number of transects and one-way ANOVAs on the effect of behaviour on the number of cats observed per transect. Activity Ranges (ARs) are presented as the average AR for dogs and cats where n = number of dogs and cats respectively and a one-way ANOVA on the effect of species on AR. Distance travelled is presented as the mean distance (metres) travelled per 24 h, per day and per night, where n = number of observations (number of collared animals) and mixed-effect models (M-EM) on the effect of species on the distance travelled.

Parameter/Statistical Test	Mean ± SD	n	DF	F	*p*
Transect					
April					
Roaming	0.5 ± 0.61	18			
Nonroaming	4.66 ± 3.16	18			
ANOVA (behaviour v survey)			1	30.1	<0.001
June					
Roaming	0.5 ± 0.85	18			
Nonroaming	2.9 ± 1.73	18			
ANOVA (behaviour v survey)			1	28.82	<0.001
Activity range (AR)					
Dog	12.15 ha ± 12.19 ha	4			
Cat	1.36 ha ± 1.42 ha	6			
ANOVA (AR v Species)			1	4.901	0.057 *
Distance Travelled per 24 h					
Dog	1233.06 m ± 545.43 m	20 (4)			
Cat	703.14 m ± 348.12 m	29 (6)			
M-EM ^#^ (Distance v Species)			1, 8	5.11	0.054 *
Distance Travelled per day					
Dog	949.68 m ± 342.82 m	20 (4)			
Cat	455.42 m ± 238.11 m	29 (6)			
M-EM ^#^ (Distance v Species)			1, 7	7.67	0.028
Distance Travelled per night					
Dog	790.5 m ± 255.86 m	20 (4)			
Cat	585.1 m ± 161.24 m	29 (6)			
M-EM ^#^ (Distance v Species)			1, 8	9.55	0.015

* Non-significant, ^#^ Mixed-effect Model.

**Table 3:** Timing of activity. Data is presented as the vector time in which: cats were observed roaming and nonroaming, where n = number of observation; dogs, cats and wildlife were observed roaming on remote-sensing cameras, where n = number of observations and dogs and cats were recorded roaming by GPS loggers, where n = the number of collared animals.

Parameter	Vector Time ± SD	n	r	R	z	*p*
Transects (pooled)						
Roaming	-	18	0.25	4.58	1.17	0.32 *
Non-roaming	12:31 a.m. ± 4 h 34 min	130	0.28	36.72	10.37	<0.001
Camera						
Dogs ^a^	8:06 a.m. and 8:06 p.m. ± 3 h 52 min	50	0.62	31.46	19.80	<0.001
Cats ^b^		3				
Wildlife	11:50 p.m. ± 3 h 52 min		0.48	49.14	23.67	<0.001
Wildlife (flightless)	12:06 a.m. ± 3 h 9 min		0.65	59.21	38.96	<0.001
GPS Loggers						
Dogs	6:02 a.m. ± 5 h 6 min	4	0.10	68.71	6.99	<0.001
Cats	11:24 a.m. ± 5 h 10 min	6	0.08	58.29	4.80	0.008

* Nonsignificant, ^a^ diametrically bimodal distribution; ^b^ statistical analysis could not be performed.

## Appendix A

**Table A1:** This table shows the location, distance from the nearest household, camera number and the number of camera nights per camera and the number of species captured on each camera.

Track	Dist. (m) ^1^	Camera	Night	Dog	Owner	Cat	Wallaby	Bandicoot	Possum	Curlew	Peewee	Cockatoo	Pigeon	Magpie	Unknown
Enrail	21	34	4	0	0	1	0	5	0	0	0	0	0	0	0
Enrail	46	29	0	0	0	0	0	0	0	0	0	0	0	0	0
Enrail	74	12	4	8	4	1	1	0	0	0	0	2	0	0	1
Enrail	149	28	4	4	10	1	0	0	0	1	0	0	0	0	0
North West	247	14	2	5	2	0	3	0	0	0	0	0	7	0	0
West	419	4	6	8	0	0	1	0	0	0	0	0	0	0	0
North West	432	22	2	4	0	0	1	0	0	0	0	0	0	0	0
North	502	38	0	0	0	0	0	0	0	0	0	0	0	0	0
West	543	35	2	0	0	0	0	0	0	0	0	0	0	0	0
North West	602	24	2	4	0	0	0	2	0	0	0	0	0	0	0
North	678	1	2	6	3	0	0	0	0	3	0	0	0	0	0
West	776	11	2	0	0	0	1	0	0	0	0	0	0	0	0
North West	792	26	2	2	0	0	1	0	0	0	0	0	0	0	0
North West	911	9	1	0	0	0	1	0	0	0	2	0	0	0	0
North	914	19	5	11	1	0	1	0	0	0	0	0	0	0	3
West	1005	18	6	4	0	0	0	0	0	0	0	0	0	0	0
North	1133	15	2	3	0	0	1	0	1	0	0	0	0	0	0
West	1166	30	6	3	0	0	1	0	0	0	0	0	0	0	0
West	1273	7	6	4	0	0	0	0	0	0	0	0	0	0	0
North	1358	31	2	4	0	0	0	0	0	0	0	0	0	0	0
West	1411	40	6	6	0	0	2	1	0	0	0	0	0	0	0
North	1507	21	4	0	0	0	4	0	0	0	0	2	0	0	0
West	1575	13	6	2	0	0	3	0	0	0	0	0	0	0	0
North	1659	3	6	0	0	0	2	2	1	4	0	0	0	0	1
North	1804	25	5	0	0	0	7	0	2	14	0	0	0	0	5
West	1830	36	6	5	0	0	0	0	0	1	0	0	0	0	1
North	2034	27	5	0	0	0	6	0	0	20	0	0	0	0	0
West	2116	16	6	0	0	0	0	0	0	0	0	0	0	17	0
North	2248	8	6	0	0	0	0	0	0	2	0	0	0	0	0

^1^ Distance (in metres) between the camera and the nearest household.

**Table A2:** This Table shows the ID, demographics, device deployment length, accumulated distance travelled and vector angle and length for each animal with an igot-U GPS logger attached to their collar.

Device	ID	Sex ^1^	Deployed ^2^	Activity Range (Ha)	Distance/24 h (m) ^3^	Distance/Day(m) ^4^	Distance/Night(m) ^5^	Vector Angle	Vector Length
G–4	Cat 4	FE	6	2.47	9684.03	5324.36	4850.99	141.14	0.21
G–7	Cat 7	FE	5	0.15	541.19	144.46	541.19	187.96	0.22 *
G–8	Cat 8	FE	6	0.53	1547.51	274.44	1024.63	269.25	0.32 *
G–9	Cat 9	MN	5	3.61	1740.87	0	1335.44	235.28	0.81
B–5	Cat 12	FS	5	1.31	2862.07	1847.43	1271.96	112.35	0.19 *
K–1	Cat 15	FE	4	0.10	635.32	753.42	249.08	144.22	0.31
K–5	Dog 2	FS	5	1.23	4479.17	4479.17	444.93	124.30	0.57
K–11	Dog 3	ME	6	8.41	8425.57	5285.96	4602.13	0.19	0.22
	Dog 3 ^#^			32,272.64					
B–14	Dog 5	ME	3	9.35	2455.74	2205.27	1646.5	75.34	0.14 ^#^
B–20	Dog 6	FS	4	29.61	14,568	9146.9	7394.83	44.71	0.50

^1^ FE: Female Entire, FS: Female Spayed, ME: Male Entire, MN: Male Neutered. ^2^ Number of days device was deployed. ^3^ Accumulated distance travelled per 24 h (midnight to midnight). ^4^ Accumulated distance travelled per day (sunrise to sunset). ^5^ Accumulated distance travelled per night (sunset to sunrise), ^#^ Value incorporates vehicle trip of Dog 3 to another settlement and his activity range whilst there, * Nonsignificant.
